# Building forward better—An exploration of nutrition practices, food choice, and coping behaviors among Kenyan adolescents during COVID‐19: Experiences and program implications

**DOI:** 10.1002/fsn3.3184

**Published:** 2022-12-27

**Authors:** Justine A. Kavle, Patrick Codjia, Constance Gathi, Brenda Ahoya, Lacey Ramirez, Stacy Katua, Florence Mugo, Laura Kiige

**Affiliations:** ^1^ Kavle Consulting, LLC Washington District of Columbia USA; ^2^ UNICEF Kenya Nairobi Kenya; ^3^ Kavle Consulting, LLC Nairobi Kenya; ^4^ Ministry of Health, Division of Nutrition and Dietetics Nairobi Kenya

**Keywords:** adolescent nutrition, COVID‐19, dietary diversity, food insecurity, physical activity

## Abstract

This implementation research study sought to examine the impact of the COVID‐19 pandemic on adolescent nutrition practices and related behaviors in Nairobi and Uasin Gishu Counties, Kenya. Eight focus group discussions (FGDs) were conducted with adolescents 10–19 years of age, in‐depth interviews with 10 health facility providers, and a combination of FGDs (*n*‐4) and key informant interviews with government stakeholder and implementing partners (*n* = 9). During the pandemic, adolescents tended to avoid commonly consumed junk foods, in favor of “*immune boosting*, *protective*” foods. Widespread unemployment and reductions in parental income rendered some food items such as meat, eggs, and fruits unaffordable for families of adolescents. Adolescents relayed experiences of skipping meals and reducing the amount and variety of foods consumed. Adolescents also described employing strategies such as working in the informal sector and selling personal items to support families financially, in response to rising food insecurity. School closures mandated during the pandemic likely contributed to reductions in overall physical activity. To improve the diets of adolescents, programs should build on the healthy mindset brought on by the pandemic, while strengthening, targeting, and improving access to social protection measures and agricultural initiatives for vulnerable families with adolescents to cushion them from rising food insecurity as an effect of COVID‐19. Building practical adolescent life skills to encourage healthy nutrition actions will also be key to building forward from the COVID‐19 pandemic in Kenya.

## Graphical abstract

Adolescents relayed experiences of skipping meals and reducing the amount and variety of foods consumed. Adolescents also described working in the informal sector and selling personal items to support families financially, in response to rising food insecurity. School closures mandated during the pandemic likely contributed to reductions in overall physical activity.

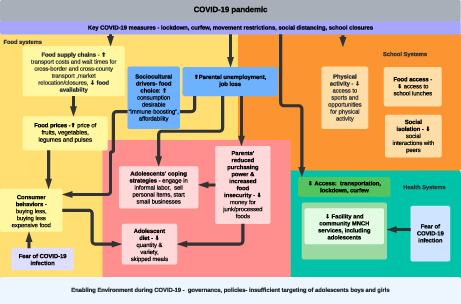

**Key messages**

1. During the COVID‐19 pandemic, adolescents described skipping meals and reduced the quality and quantity of foods consumed, largely due to parental job loss and families' inability to afford some foods, such as animal‐source foods and fruits.
2. In response to rising food insecurity, some adolescents described experiences of selling personal items, working jobs in the informal sector, or starting businesses to support household incomes to buy food.
3. The COVID‐19 pandemic prompted some adolescents to make better food choices due to the belief that immune‐boosting foods provided protection against COVID‐19 and due to reductions in parents' purchasing power to afford unhealthy junk foods.
4. School closures due to the COVID‐19 pandemic notably reduced reported physical activity.

## 1 INTRODUCTION

Adequate nutrition is critical to support accelerated physical growth (i.e., stature), timely onset of puberty, changes in body composition (i.e., bone, muscle, and fat), and continued development of the maturing brain in the transition from childhood to adolescence (Norris et al., 2022). Yet, in low‐resource settings, adolescent boys and girls may be vulnerable to multiple forms of malnutrition, including undernutrition and obesity, due to poor‐quality diets consisting of high‐fat, high‐sugar, and/or energy‐dense processed foods, which can threaten healthy growth and alter brain function (Lowe et al., 2020; Norris et al., 2022).

Across sub‐Saharan Africa (SSA), the COVID‐19 pandemic has put adolescents and other vulnerable groups (i.e., women of reproductive age and children under 5 years of age) at risk for micronutrient deficiencies and deteriorating nutritional status, in the face of rising food insecurity and worsening economic strife (Cluver et al., 2020; UNICEF, 2021; M. Wang, 2021). In the SSA region, children and adolescents under 19 years of age bear the brunt of undernutrition (i.e., wasting and stunting), sickle cell disease, and other chronic conditions and illnesses, such as pneumonia, diarrhea, malaria, tuberculosis, and HIV (Coker et al., 2021). Country‐mandated lockdowns intensified children and adolescents' vulnerabilities to noncommunicable diseases (NCDs) in SSA by reducing household income and limiting access to health services (Nachega et al., 2022). This is particularly concerning given cohort data reveal an association between NCDs and substantially higher morbidity and mortality rates among children and adolescents hospitalized with COVID‐19 in six SSA countries, including Kenya, in comparison to non‐SSA country settings (Nachega et al., 2022).

In Kenya, about one‐quarter of the population (47 million) are adolescents, 10–19 years of age (UNFPA, 2017). While the Africa Center for Disease Control and Prevention noted that children aged 0–14 years comprise only 2.1% of confirmed cases, the detrimental effects of COVID‐19 have affected various dimensions of adolescent health, yet have not been well characterized (Africa CDC, 2020). The effects of COVID‐19 on adolescent health and nutrition practices and related behaviors remain limited. While a few studies have examined knowledge and awareness of COVID‐19 among adolescents in sub‐Saharan Africa, a paucity of information exists on adolescent's experiences during the COVID‐19 pandemic in terms of household food insecurity, food consumption patterns, physical activity, and coping strategies (Addae, 2021; Marotta et al., 2021). To our knowledge, this is one of the first in‐depth explorations of these themes using qualitative methodologies in sub‐Saharan Africa and specifically in Kenya, which was largely collected in‐person. The objectives of this study are: (1) to understand how the COVID‐19 pandemic affected adolescents' experiences of food insecurity, in terms of food choice, consumption patterns, and related coping behaviors, in Nairobi and Uasin Gishu Counties, Kenya; and (2) provide program considerations for future pandemic preparedness based on the adolescents' experiences during COVID‐19 in Kenya.

## 2 MATERIALS AND METHODS

### 2.1 Study design and site

Consensus from key government, partner organization, and academic stakeholders in the country, through various national technical working groups (i.e., MIYCN Technical Working Group, Research Technical Working Group; Ministry of Health, Kenya), was garnered to determine criteria for study site selection. Criteria for study site inclusion were as follows: (1) counties with the highest COVID‐19 burden based on MOH data on COVID‐19 infections/cases; (2) one urban and one rural county; (3) one county which experienced at least one government lockdown versus. one county that did not experience a lockdown during the course of COVID‐19 pandemic; and (4) counties which included persons of low socioeconomic status who resided in informal settlements were selected in consultation with the government officials, and Ministry of Health country data. Ultimately, Nairobi and Uasin Gishu counties had the highest burden of COVID‐19 cases in Kenya (Ministry of Health (MoH), Kenya, 2021) and met study criteria for selection. In Nairobi County, urban Embakasi East and Kibra subcounties are comprised of informal settlements in the country. Kibra subcounty had the third highest number of COVID‐19 infections in the county, which was notable. In Uasin Gishu County, rural Ainabkoi, and Turbo subcounties, accounted for almost half of the COVID‐19 infections within a county which had not experienced a mandated COVID‐19 lockdown.

### 2.2 Data collection

Fieldwork occurred between August and September 2021, and country caseloads of COVID‐19, per available Ministry of Health data were 36,479 and 10,331 for August and September, respectively (Ministry of Health – Republic of Kenya, 2022). Data collection for adolescents was part of a larger implementation research study collecting qualitative information from pregnant and lactating women on dietary intake, access, and use of health services, while also examining perspectives from health workers, community health volunteers, and food vendors on effects on the health and food systems (Ahoya et al., 2022). While study participants were part of this larger implementation research study, adolescent boys and girls were selected as an independent sample via opportunistic purposeful sampling (Patton, 1990). Data were largely collected in‐person with adolescents, with some virtual data collection for stakeholders, due to the COVID‐19 pandemic. Inclusion criteria for study participation included adolescent boys and girls 10–19 years of age, residents of study sites for at least a 3‐year period, and nonpregnant and/or non‐lactating individuals (i.e., adolescent girls only).

Within each study site, local community health volunteers associated with the nearest health facility offering maternal, newborn, and child health services supported the identification of adolescents for study enrollment. In‐depth interviews (IDIs) with facility health workers and key informant interviews (KIIs) and small focus group discussions (FGDs) with key stakeholders (i.e., national and county government officials and key focal points from implementing partner organizations) explored topics such as integration of adolescent nutrition into health services, adolescent food choice and consumption, related adolescent behaviors in response to food insecurity and school closures, and physical activity during the COVID‐19 pandemic. FGDs with adolescents explored the following topics: effect of the pandemic on beliefs and perceptions of foods, decisions around food choice, school attendance, physical activity, and coping strategies during the pandemic.

Eight face‐to‐face FGDs were conducted with 54 adolescents, 10–19 years of age, and IDIs with 10 facility health workers (i.e., 6 in‐person and 4 virtual). Virtual data collection, using the Zoom platform, was used for all 18 stakeholders, which was comprised of national and county government officials and implementing partners (except 2 in‐person FGDs, see Table 1). Four small FGDs were carried out with nine county/subcounty stakeholders working on maternal, adolescent, infant, and young child nutrition (MAIYCN) programs, KIIs with national‐level government nutrition officials (*n* = 4), and KIIs with government officials and implementing partners working in adolescent programming at national and subnational levels (i.e., county and subcounty) (*n* = 5). COVID‐19 precautions were also followed during face‐to‐face data collection—including donning of face masks during interviews and FGDs, use of hand sanitizers and handwashing with water and soap, as available, and social distancing of 3 m between interviewer and study participant(s).

Local ethical approval was obtained from the Institutional Ethics Review Committee of Masinde Muliro University of Science and Technology, Kenya, and country‐based research license was obtained from National Commission for Science Technology and Innovation. Verbal informed consent was obtained from study participants.

### 2.3 Data management and analysis

Trained interviewers audio recorded all IDIs and FGDs, which were translated and transcribed verbatim from Swahili into English. The quality of transcriptions was checked for accuracy and completeness against the audio recordings by the Kenyan research team members, alongside local trained transcribers. The researchers (JK, CG, BA , and LR) conducted a preliminary review of the data by reading a subset of the transcripts to create an initial codebook that included major themes and subthemes that emerged from the data. The researchers (JK, CG, BA, and LR) then coded a subset of the transcripts and discussed, and a consensus was reached among research team members regarding any discrepancies in coding. The codebook was further refined and used for coding (see Supplementary File S1). Once coding was complete, researchers looked independently at a subset of transcripts for verification of the themes in the codebook and to confirm any additional emerging concepts. Transcripts were further reviewed and triangulated with corresponding field data collection forms. All transcripts were coded using Dedoose online software. Each subtheme was then summarized, and illustrative quotes were selected (see Table 2).

## 3 RESULTS

**FIGURE 1:**
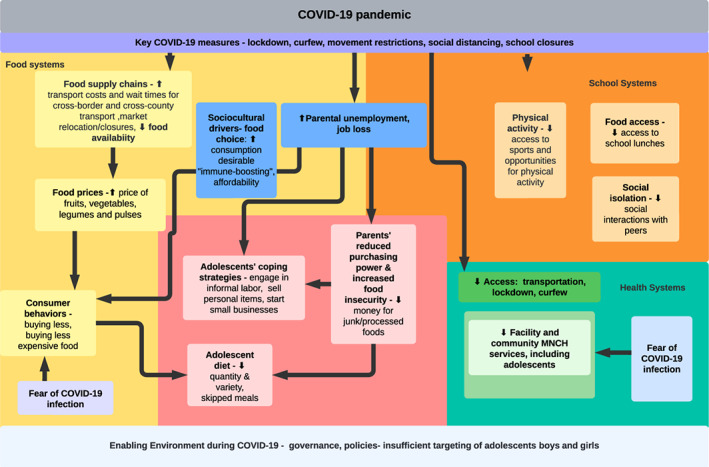
Conceptual framework on the impact of COVID‐19 pandemic on adolescents, via aspects of health, food, and school systems

Figure 1 presents the conceptual framework which characterized the impact of COVID‐19 on aspects of adolescent nutrition practices and coping strategies in Kenya. Based on key findings from this study, key factors which affected dietary practices (i.e., drivers and beliefs surrounding food choice and consumption) were added to the framework. The conceptual framework illustrates how the COVID‐19 pandemic affected food, school, and health systems, taking into account job loss, sociocultural drivers, fear of COVID‐19 infection, and rising food insecurity, which negatively affected adolescent nutrition (i.e., reduced meal frequency and quality of diet) (See Figure 1). This study was part of a larger overall study examining the effect of COVID‐19 on nutrition practices of pregnant women, breastfeeding women and their infants, health service delivery, and key aspects of food systems (i.e., food vendors).

### 3.1 Characteristics of study participants

Eight FGDs were conducted separately with 54 adolescents, stratified by age and gender, to ensure adolescents could discuss topics openly with their peers (Table 1). Half of the adolescents were 10–14 years of age, while the remaining were 15–19 years of age. Twenty‐nine adolescents were from urban informal settlements (i.e., slum areas) in Nairobi County, while twenty‐five adolescents resided in rural, agricultural areas in Uasin Gishu County. Each FGD had between five and nine adolescents, who were a mixture of those identified as attending school prior to the pandemic, while others (i.e., older) had begun working and no longer attended school. FGDs were also conducted with a variety of stakeholders at national and subnational levels (i.e., county and subcounty) working as nutrition‐specific (maternal, adolescent, infant, and young child programming) and as implementing partners (nongovernmental organizations).

**TABLE 1:** Characteristics of study participants, by methodology and study site, Nairobi and Uasin Gishu Counties

	Nairobi County		Uasin Gishu County			All
	Adolescents focus group discussions (FGDs) *n* = 29	In‐depth interviews (IDIs)^a^ *N* = 5	Adolescents FGDs *n* = 25	IDIs *n* = 5	Stakeholder FGD participants^b^	Stakeholder key informant interviews *n* = 9
Age—Adolescents						
15–19 years	13	‐	13			
20–24 years	16	‐	12			
Socioeconomic Status—Adolescents						
Informal settlements/urban	29	‐	‐	‐		
Rural	‐	‐	25	‐		
Occupation—Health providers and Stakeholders						
Nurse		5	‐	5		
NGO/implementing partner focal point						1
National government officials						5
County government officials					9	3
Total	29	5	25	5	9	9

^a^ Facility Health Worker.

^*b*^ Four FGDs: two2 FGDs conducted in‐person and two FGDs virtually.

The themes that emerged from the qualitative analysis are discussed in Table 2.

**TABLE 2:** Summary of dominant themes by study participant group, Nairobi (NC) and Uasin Gishu (UGC) Counties

Themes	Young adolescent boys and girls, 10–14 years of age (*n* = 26)	Older adolescents boys and girls, 15–19 years of age (*n* = 28)	Stakeholders (*n* = 18)	Facility health workers (*n* = 10)
Adolescents skipped meals and consumed smaller portions— in response to household food insecurity	“Before, we were being served with big plates and they served them full to the brim [at school]. But nowadays, they serve small food with small plates, and we don’t get satisfied.”—Adolescent FGD Nairobi County	R3: “In the morning, black tea with mandazis in case its available. If it's not, we take it with leftovers from the previous night. Lunch is uncertain. We may or may not eat. Then supper, we take whatever is available and life continues.” R4: “In the morning, we take tea with bread then skip lunch and wait for supper to take ugali with kale mixed with avocado….and then wait for the following day [to eat].” R6: “In the morning, we usually wake up to take tea with 2 mandazis. Then lunch, it's by chance. We may or may not eat. Then supper, we take what's available… although mostly it's served in low quantity to be enough for my small siblings and I.” R7: “Whatever is there is what we take, whether it's in low or high quantity.”—Adolescent FGD Nairobi County	“People lost jobs, so they didn’t have enough money to buy the food that they are used to. So, of course they had to reduce the number of times they eat the food and also reduce the types of foods they buy. If they normally buy maybe 3 types of food for a meal, I believe they reduced the number maybe to just now one type of food per meal or just two.”—Key Informant, Uasin Gishu County	N/A
Reduced adolescent physical activity because of COVID‐19 lockdown and school closures	“You see, as before, we [would] wake up around 6 a.m… You see, we would run up to around 8 a.m. But with corona… I don’t remember that last day I did that.”—Adolescent FGD Nairobi County	“Boys here love playing football, but now getting an opportunity to play isn’t easy – not unless you play it at your home compound with a few of your friends. But before corona, we could go to play at the community ground. But now since the pandemic, we no longer play there because of restrictions on social gatherings.”—Adolescent FGD Nairobi County	“Remember people were being told to stay at home, irrespective of the age group that you're talking about here. So, the issue with staying at home [is that] physical activity was reduced. Remember we also used to tell them even the children are not supposed to be in those groups playing with their neighbors and all that because everybody feared the disease.”—Stakeholder FGD Nairobi County	“In our community, we did have games and activities. We used to offer challenges between churches to churches, church to hospital, community versus another community, which was being handled by the hospital committees. But since COVID‐19 started, physical activity was minimized, so we had to minimize contact because no one can play in a field with a mask. You cannot run in a mask.”—Health Worker, Uasin Gishu County
Adolescents and/or parents worked “casual” jobs (i.e., fetching water and selling vegetables) to supplement household income and purchase food for the family	“[My parents] lost their jobs, and they were forced to wash clothes for households to get [money].”—Adolescent FGD, Nairobi County	“Let me use myself as an example… I sold like my phone, I sold some of my shoes, my trouser … because during that time my parent had nothing completely, he had even come to stay home… so when I sold those things I bought vegetables, sugar, tea leaves… and I told her not to panic because we don’t have… the little that we get is what we will eat.”—Adolescent FGD, Uasin Gishu County	“One of the measures that was put in place was to lock down Nairobi. Movement into and out of Nairobi was restricted, and for many people, this really affected their livelihood because some people could not get food from upcountry and the like. And [for] some people, even going to work became an issue. But at the initial point, what I can report is mostly people lost jobs. Remember, the hotels were locked, the curfew hours were mainly reduced to 7 p.m. So, there are many challenges that led to food insecurity, which was a big issue in Nairobi, affecting the whole population—Stakeholder FGD, Nairobi County	“Maybe their parent lost their job. So, there is no way they could get back to school, so they had to seek alternatives, or else they had to drop out. Maybe if it is the male, they went to at least to do some kind of daily work at least to meet their basic needs.”—Health Worker, Nairobi County

#### 3.1.1 COVID‐19 guidance on implementation of adolescent nutrition activities was limited

Facility health workers relayed that they were not trained on measures to address adolescent nutrition during the COVID‐19 pandemic and only received general, cross‐cutting guidance. Despite the dissemination of nutrition in COVID‐19 guidance, the majority of health workers described not being aware of any nutritional guidance or support for adolescents during COVID‐19 pandemic and did not feel *“well‐placed to respond”* to adolescent nutrition issues, such as dietary practices and physical activity during COVID‐19. Stakeholders, key informants, and health workers reported that there were no specific nutrition services during COVID‐19 directed to adolescents 10–19 years of age, apart from services targeted to pregnant and lactating adolescents as part of the routine ANC/PNC package

#### 3.1.2 Adolescents tended to eat healthier and avoid commonly consumed fast foods during the COVID‐19 pandemic

Adolescents' held food preferences for *chapatti* (i.e., unleavened flat bread made from wheat flour), fried/fast foods, *bhajia* (i.e., potatoes dipped in flour batter and fried in vegetable oil), meat, bread, and sweets. Yet, the majority of adolescents consumed nutritious foods during COVID‐19, such as kale, ugali, beans, and *githeri* (i.e., meal made up of a mixture of maize and bean), and traditional vegetables (see Table 3). Most adolescents had positive perceptions of these foods as “*healthy*” and “*energy‐giving*” as well as “*cheap*” and “*easily available”* in their communities, which drove food choice.

**TABLE 3:** Food items and reasons for consumption among adolescents, 10–19 years of age, Nairobi and Uasin Gishu counties, Kenya

Category	Food items	Reasons for consumption	Illustrative quotes
Preferred foods eaten by adolescents prior to COVID‐19	Ugali, chapatti, Fried/fast foods (i.e., chips and bhajia), meat (i.e., beef with rice), bread, and fruit	Parents could afford to buy for adolescents. Adolescents liked to consume these foods.	“Before corona came, our parents would go for work and when they get money, they buy us meat, rice, and chapatti. And now that there is COVID‐19, getting those things is very hard.” —Adolescent FGD, Uasin Gishu County “Before COVID, I used to eat chips thrice in a day but now maybe once per week because I do not have that money to buy them.”—Adolescent FGD, Nairobi County
Commonly consumed foods during the COVID‐19 pandemic	↑ Ugali ↑ Green leafy vegetables (i.e., kale) ↑ Traditional vegetables (i.e., sagas) ↑ Black tea, githeri, ↑potatoes,	Cheap/ “*don’t have enough money*,” available, energy‐giving, healthy	“Since covid started, the foods that we used to get for breakfast [were] like maybe tea, eggs, and bread. [For] lunch maybe ugali, and for supper, we eat ugali and beef. But now, things are no longer the same with COVID. We are forced to take tea and mandazi for breakfast and wait for supper, which is ugali and kale every day.”—Adolescent FGD, Nairobi County
Foods avoided and reduced during the COVID‐19 pandemic	↓Juice, ↓Soda ↓Cakes ↓Biscuits ↓Mandazi ↓Chapatti ↓Bread ↓Vegetables (cabbage) ↓Beans ↓Rice ↓Milk ↓Meat ↓Fruits ↓Sweets ↓Eggs	“*Don’t have enough money*” “*Did not boost immunity/increases COVID‐19 infection*” (soda only), unhealthy High prices of foods and loss of jobs/lack of money	“Before COVID‐19, [powdered] juice would be bought for us [by our parents], but right now it is very rare for us to drink even soda. [Before], we could even buy a crate of soda, but right now it costs 700, so we cannot afford to buy soda.” —Adolescent FGD, Uasin Gishu County *“*Nowadays, getting foods like bhajia and chips is not easy since the price of cooking oil and potatoes went up because of COVID, unlike before the pandemic when we could easily access those foods at cheaper prices.”—Adolescent FGD, Nairobi County “We were eating chapatti, beans, and fruits every week because then it was cheap, but now it is very expensive because a big packet of flour goes for 150 [Kenyan shillings], so you would rather buy potatoes and eat it twice. But chapatti is very rare.” —Adolescent FGD, Uasin Gishu County

*“Before Corona [COVID‐19]*, *my parents had the means to bring us every kind of food. That time we used to eat rice twice a week*, *Ugali once*, *and chapatti… but since corona came*, *we are eating Ugali every day because that is the meal that is available.”*—Adolescent FGD, Uasin Gishu County

#### 3.1.3 Skipping meals was commonplace among adolescent boys and girls

Nearly all adolescents expressed frustration with being “*forced* ” to skip meals, such as lunch, in response to pandemic‐related reductions in household food availability. Some adolescents relayed that “*getting food is very difficult.”* COVID‐19 largely limited their diets to *ugali* (i.e., a local porridge made from stiff maize flour) and green leafy vegetables (i.e., kale) due to parental job loss and the unaffordability of certain foods such as meat, eggs, and bread as expressed by some adolescents:

*“When Corona started* *is the day problems came… because for us we forgot about lunch. In our home, we don't have lunch and sometimes we take tea without anything else…. And you know, we had lockdown and jobs reduced. So*, *in my experience*, *Corona has affected me badly…”*—Adolescent FGD, Uasin Gishu County

#### 3.1.4 Adolescents often reduced quantities of food consumed during the pandemic, including fast foods and sweets

Some adolescents expressed eating “*what is available”* (i.e., leftovers from the night before) due to lack of money from their parents (see Table 3). Adolescents also reported consuming reduced quantities of food to ensure that there was enough food available for younger children in the family in both Nairobi and Uasin Gishu Counties.

*“Right now*, *there is no money*, *so you just share the little among yourselves even if it is vegetables and ugali. If you don't have money… you just cook a little ugali and some vegetables and share it among everyone.”—* Adolescent FGD, Uasin Gishu County

*“In the morning*, *we usually wake up to take tea with 2 mandazis. Lunch is by chance*, *we may or may not eat. Then*, *supper, we take what's available*, *although mostly it is served in small quantities to be enough for my small siblings and I.”*—Adolescent FGD, Nairobi County

In both counties, some adolescents discussed how certain foods became very expensive during the pandemic (e.g., fruits, cabbage, tomatoes, eggs, meat, beans, green bananas, and cooking oil), which decreased the variety of foods that they consumed during the pandemic. Some adolescents also mentioned increased consumption of black tea instead of tea with milk during COVID‐19. Urban adolescents also reported the relocation of their families to their rural homes where there was perceived greater food availability, as a coping strategy in response to food insecurity.

#### 3.1.5 Some adolescents consumed “immune‐boosting” foods, claiming they were protective against COVID‐19

Nearly all adolescents held beliefs that specific foods, such as green leafy vegetables, ginger, fruits, garlic, and lemon, could provide a *“*strong immunity” against COVID‐19 infection, and often consumed a lemon–ginger–honey concoctions alongside traditional herbs. A few adolescents also believed that sugary foods should be avoided, as these foods were viewed as “*not protective*” against COVID‐19 infection, as relayed in the quote below.

*“Like [certain common foods like] chapatti*, *mandazi* (i.e. leavened deep fried dough made from wheat flour) and *special foods such as soda [*we normally have*]*, *but right now no*, *[we don't eat them]. To prevent COVID‐19 you don't need sugary foods because they don't protect against COVID‐19."*—Adolescent FGD, Uasin Gishu County

#### 3.1.6 Some adolescents reduced engagement in sports and physical activity

As a consequence of school closures, lockdowns, and social distancing, adolescents discussed less engagement in sports and physical activity, such as ball games and running.

*“[Before COVID‐19]*, *when we were at school*, *we would go for games. But when COVID‐19 started, we were just at home*, *so we weren't doing exercise.”*—Adolescent FGD, Uasin Gishu County.

#### 3.1.7 Adolescents supported their families financially in response to household food insecurity

Older adolescent boys and girls worked to financially support their families by starting their own small businesses, either selling charcoal or food or engaging in casual labor like farm work. Focus group discussants described their families' financial struggles, which went hand in hand with food insecurity, during the pandemic, as illustrated below:

*“The only time when we eat to our satisfaction is when my siblings and I contribute some money to buy something like sardines to eat with ugali or at times my mother would give me something like cooking pans to sell and that's how we survived because my mother is jobless.”* —Adolescent FGD, Nairobi County

Some adolescents were also forced to sell their personal belongings and clothing items to support their families to purchase food for the household.

*“Let me use myself as an example… I sold like my phone*, *I sold some of my shoes*, *my trouser … because during that time my parent had nothing completely*, *she had even come to stay home… so when I sold those things I bought vegetables*, *sugar*, *tea leaves… and I told her not to panic because we don't have… the little that we get is what we will eat.”—* Adolescent FGD Uasin Gishu County

## 4 DISCUSSION

This study sought to gain an understanding of adolescents' lived experiences of food insecurity via food choice, consumption, and coping strategies and provide program considerations to guide future pandemic preparedness based on the findings reported here. Adolescents expressed reluctantly reducing their meal frequency, quantity, and diversity as a consequence of parents' inability to afford foods routinely consumed in the household—as a result of the pandemic.

Importantly, most adolescents felt the brunt of parental job loss and in response worked casual labor, started their own businesses selling food or other goods, and sold personal items in an effort to support their families, in response to rising household food insecurity. Adolescents expressed frustration with school closures, which reduced available play spaces and subsequent physical activity. School closures during the COVID‐19 pandemic reduced physical activity for most adolescent boys and girls.

Adolescents described greater financial losses when family members and older adolescents themselves experienced loss in employment from the informal sector in this study. A few past studies in Ethiopia and Kenya noted that declines in household income were often met with reductions in food consumption as a coping mechanism (Alderman et al., 2020). In an online survey among 2156 Kenyan youth, 18–35 years of age, half of the participants had a significant decline in income due to unemployment or job loss from informal sector, while one‐third reported increased food prices which rendered local foods less affordable (Karijo et al., 2021).

Importantly, this study revealed that these losses in family income fueled food insecurity as skipping meals was commonplace among adolescent boys and girls, 10–19 years of age, during the COVID‐19 pandemic. A previous study conducted with Kenyan adolescent girls, 15–19 years of age, and young women, 20–24 years of age, reported that most (59%–72%) skipped meals during the COVID‐19 pandemic, with the highest proportion in Nairobi County (Karp et al., 2021). Moreover, one‐third to half of the study households experienced total and partial income loss, respectively (Karp et al., 2021). Similar findings were noted in a multicountry study with children and young adolescents, 9–13 years of age, in Nigeria, Tanzania, and Sierra Leone, as most families expressed difficulties in getting or purchasing food, especially among larger households with extended family members (Kallander et al., 2021). Previous studies reveal that food insecurity and hunger were significant challenges facing youth. For example, 62% of Ugandan adolescent boys and young men relayed difficulties in affordability and attaining dietary diversity and *“balanced diets”* (Matovu et al., 2021). While prior to pandemic, adolescents discussed routine consumption of unhealthy “*junk*” foods in this study, some adolescents also relayed that fried food, such as *bhajia* and chips (i.e., local snacks made of fried potatoes), were higher in cost and considered “*unnecessary* “and unaffordable by parents and adolescents, which curbed intake of these unhealthy foods during the pandemic. Notably, adolescents had a tendency to eat healthier, despite consuming less variety of foods—which may have also been attributed to parents' economic situation to afford desired unhealthy foods.

This study also revealed stress and fear experienced by adolescents in response to COVID‐19‐fueled food insecurity and financial difficulties, which often forced adolescents to sell personal items and work informal labor jobs to support their families, and relocation of urban families to rural homes to mitigate the effects of COVID‐19 food insecurity. A few studies echoed our findings, pointing to widespread parental job loss which often translated to use of multiple strategies by families to increase overall household income (Decker et al., 2021; Karijo et al., 2021). An online phone‐based survey in Nairobi revealed that losses in family members' earnings resulted in borrowing loans and moving into smaller homes with extended family members to afford basic needs, such as rent and food (Decker et al., 2021). Formative research conducted prior to the pandemic in Kenya, Tanzania, and Uganda shows that adolescents often carried out income‐generating activities via heavy manual labor at a detriment to their health (Hall et al., 2019; WFP, 2018, 2019). Adolescents' engagement in these activities may have worsened during the COVID‐19 pandemic.

Communities that rely on adolescents for income to purchase food may limit their opportunities for future education, even prior to COVID‐19 (Neufeld et al., 2022). Our data indicate that COVID‐19‐mandated school closures likely negatively impacted healthy adolescent behavior, such as physical activity, due to lack of access and/or planned activities. Past studies corroborate our findings of reductions in physical activity and involvement in sports reported by adolescents during the COVID‐19 pandemic. Phone survey data from Burkina Faso, Ethiopia, and Nigeria pointed to a 38% overall decrease in physical activity (D. Wang et al., 2021), while self‐reported reductions in physical activity ranged from 12% to 45% in urban Nigeria and urban Ethiopia, respectively (Wang et al., 2021). In Kampala, Uganda, more than half of the participants 10–19 years of age reported an increase in sedentary lifestyle with excessive television watching during the pandemic (Matovu et al., 2021). Moreover, adolescents who experienced school closures in Ethiopia, Burkina Faso, and/or Nigeria ate fewer staple foods (9%–54%), pulses (29%–49%), fruits (18%–41%), vegetables (3%–34%), and animal source foods (11%–29%) (Wang et al., 2021).

Other previous research studies have linked school disruptions and food insecurity to additional negative consequences that impinge on quality of adolescents' lives, including increased feelings of anxiety and stress, loss of interest in studying, feeling “*stuck*,” and loss of purpose in Bangladesh, Kenya, and South Africa (Gittings et al., 2021; Karp et al., 2021; Oakley et al., 2020). Recent evidence from Nairobi, Kilifi, and Kisumu Counties noted that economic strains led to conflict and tensions in Kenyans’ households (Karp et al., 2021). Furthermore, a recent systematic review illuminated that COVID‐19‐induced social isolation and loneliness experienced by adolescents increased the risk of depression, which reverberated at 0.25 and 9 years later, impacting adolescent well‐being (Loades et al., 2020). While not well‐characterized in SSA, these issues merit further exploration based on the lived experiences of young people.

While the stress of the COVID‐19 pandemic brought additional work burdens in the lived experiences of food insecurity described by adolescents, most adolescents expressed a “*healthy eating*” mindset and a desire to consume healthier and “*immune‐boosting”* foods as a means of “*protection against the virus*.” Leveraging upon these instilled beliefs and experiences shaped by the COVID‐19 pandemic can be used to equip adolescents as agents of change in their communities (Neufeld et al., 2022), while recognizing that work is still needed to address the routine intake of unhealthy, processed, and/or junk foods among young persons beyond the pandemic. The Kenya Nutrition Action Plan (2018–2022) aims to: “*increase nutrition awareness and uptake of nutrition services for improved nutritional status of older children and adolescents* (*10–19 years of age*)” ‐ with strategies that include advocacy and promotion for adolescent nutrition, building stakeholder capacity on healthy diets, sensitizing communities and stakeholders, as well as promoting consumption and marketing of healthy foods and physical activity for adolescents (Ministry of Health, 2018). Yet, our data suggest that specifically targeted services and interventions for adolescents were largely absent during the COVID‐19 response. Future pandemic preparedness efforts should ensure that changes in adolescent nutrition practices, and psychosocial needs during emergencies are addressed. In Kenya, adolescents have a potential role to play by partnering with agriculture initiatives and/or youth‐led activities, which may provide additional safeguards against food insecurity, by providing education and life skills opportunities, including providing access to foods, and other skills (i.e., saving money, safe income‐generating skills, health behaviors, and eating habits) (Neufeld et al., 2022; United Nations, 2020).

## 5 LIMITATIONS

Detailed dietary data from adolescent girls and boys were not collected during this study, although foods consumed, beliefs and perceptions, and associated factors surrounding food choice and consumption were derived through qualitative methodologies. Detailed educational data from adolescents were not collected during the COVID‐19 pandemic, however, most adolescents, aged 10 to 19, attended school and relayed issues related to school closures, while older adolescents discussed working to support their families rather than attending school. Given this data collection was part of a larger study examining health and food systems and MAIYCN practices, the original aim of this study did not cover issues with school systems at length. Therefore, additional information on the nutrition provided in schools (i.e., school feeding) was not collected during the course of this study, but rather COVID‐19‐specific effects from school closures—largely from adolescents’, key informants’, and health providers' perspectives. While the COVID‐19 pandemic limited our ability to collect all data in person, importantly this study, one of the first in the region, had face‐to‐face FGDs with all adolescents, health providers, and half of stakeholders.

### 5.1 Program considerations for adolescent programming in the context of future pandemic preparedness

Build practical adolescent life skills around nutrition during prolonged school closures or when adolescents are not in school, such as safe income‐generating skills, health behaviors, and eating habits, and through backyard (i.e., “kitchen”) gardens in their families' homes to cushion against household food insecurity.

1. Strengthen design and implementation of social behavior change strategies and interventions to address unhealthy nutrition practices and associated health and coping behaviors for adolescents whose families newly face food insecurity.
2. Strengthen community‐based platforms, such as peer and community support groups, that facilitate safety, health, and nutrition information for adolescents, building upon the “*healthy eating*” mindset that began during the pandemic (Govender et al., 2020; Wang et al., 2021).
3. Improve access to targeted social protection measures to cushion families and adolescents affected by the informal work sector, including the provision of cash transfers and food subsidies. For example, consider use of support funds distributed per child or per caregiver during prolonged school closures, during times of emergency/pandemic, which can provide additional monetary support to vulnerable families, as has been carried out in South Africa (South African Government, 2020).
4. Monitor food shortages and support families to access social relief funds (Govender et al., 2020).
5. Strengthen capacity of health workers to provide adolescent‐responsive health services.

## 6 CONCLUSION

This implementation research study provided information on how the COVID‐19 pandemic shaped adolescent nutrition practices and associated coping behaviors while considering key aspects of the food, school, and health systems in Kenya. Building forward better from COVID‐19 requires addressing gaps in adolescent nutrition knowledge and awareness. Supporting community‐based education to build upon adolescents' healthy eating mindset, while addressing issues of meal skipping and quality of foods consumed—which persisted due to the pandemic—will be key. Moving forward, it will be critical to also strengthen social protection measures and agriculture initiatives, targeting families and adolescents alongside the aforementioned nutrition‐specific measures. Equipping adolescents with life skills may go far in addressing unhealthy nutrition behaviors to improve dietary intake and physical activity in the face of rising food insecurity.

## FUNDING INFORMATION

This publication is made possible through funding provided by UNICEF Kenya.

## CONFLICT OF INTEREST

Team members from Kavle Consulting, LLC were hired for a consultancy with UNICEF Kenya to carry out this study.

## ETHICAL APPROVAL

This study was approved by the Institutional Ethics Review Committee of Masinde Muliro University of Science and Technology, Kenya, and the country‐based research license was obtained from National Commission for Science Technology and Innovation.

## INFORMED CONSENT

Written informed consent was obtained from all study participants.

## Supporting information


Appendix S1.

## Data Availability

The data that support the findings of this study are available on request from the corresponding author. The data are not publicly available due to privacy or ethical restrictions.
