# Supplementary material for: Building forward better—An exploration of nutrition practices, food choice, and coping behaviors among Kenyan adolescents during COVID‐19: Experiences and program implications
Source: Food Sci Nutr. 2022 Dec 27;11(3):1441–51. doi: 10.1002/fsn3.3184 (PMC9880680; doi:10.1002/fsn3.3184)
Supplement: Supplementary file 1 — Appendix S1. [file FSN3-11-1441-s001.doc]

**Supplementary File S1. Codebook**

| **Ref.** | **Codes** | **Definition** |
| --- | --- | --- |
| *1 – Pregnant/lactating women* | | |
| 1A | Changes in diet | Description of dietary practices and ways they have changed during the COVID-19 pandemic for pregnant/lactating women. Includes types of foods eaten (including immune-boosting foods) and frequency consumed. |
| 1B | Beliefs/perceptions | Discussion of pregnant/lactating women’s attitude, beliefs, and/or perceptions of foods they might consume or avoid (e.g., description of foods believed to be healthy or immune boosting or foods believed to be unhealthy). |
| 1C | Micronutrient supplementation | Discussion of micronutrient supplements (e.g., IFAS – iron folic-acid supplementation or folic acid or other micro-nutrient supplementation / deworming) taken by pregnant/lactating women and changes to access/availability or supplements taken during the COVID-19 pandemic. |
| 1D | Advice/counselling | Discussion of advice/counselling pregnant/lactating women say they receive (e.g., advice on women’s diet, breastfeeding, foods to feed children, mental health etc. (providers/ family members). Including COVID guidance in breastfeeding |
| 1E | Coping strategies | Types of coping strategies mentioned, including skipping/reducing meals, or reducing quantity, reducing quality, preference for cheaper/available foods/ready cooked foods, use of mobile lending applications. |
| 1F | Other challenges/changes | Other challenges/changes/stressors to diet or health pregnant/lactating women experienced during the COVID-19 pandemic including gaps in psychosocial support. |
| *2 – Infants/young children* | | |
| 2A | Complementary feeding | Discussion of complementary feeding, including first foods given to infants/young children and ways that dietary practices may have been challenged or changed during the COVID-19 pandemic. |
| 2B | Beliefs/perceptions | Discussion of pregnant/lactating women’s attitude, beliefs, and/or perceptions of foods they might feed their infants or young children. |
| 2C | Acute malnutrition | Discussion of acute malnutrition including mentions of increased cases of wasting, acute malnutrition, and signs/symptoms of acute malnutrition. |
| 2D | Other challenges/changes | Other challenges/changes in diet or health infants/young children experienced during the COVID-19 pandemic. |
| *3 – Breastfeeding* | | |
| 3A | Early initiation of breastfeeding | Discussion of first feeding after birth, including initiation of breastfeeding, rooming-in, or separation of mother and infant at childbirth. Also includes mention of how the first feeding may have been influenced by the COVID-19 pandemic, including hospital policy or practices during COVID-19. |
| 3B | Exclusive breastfeeding | Discussion of exclusive breastfeeding (feeding breastmilk in the first 6 months of life) |
| 3C | Mixed feeding | Discussion of mixed feeding including breastmilk substitutes, infant formula use, early introduction of foods before 6 months (such as mixing porridge with water or giving tea). |
| 3D | Continued breastfeeding after 6 months | Discussion of continued breastfeeding after 6 months, including early cessation of breastfeeding (stopping breastfeeding for any reason), and continued use of infant formula after 6 months. |
| 3E | Beliefs/perceptions | Discussion of pregnant/lactating women’ attitude, beliefs, and/or perceptions surrounding breastfeeding and breastmilk (e.g., beliefs about not having enough breastmilk or specific foods to increase milk supply), beliefs around breastfeeding and COVID |
| 3F | Breastfeeding while COVID-19 POS | Discussion on experience or guidance received on breastfeeding after testing positive for COVID-19, including use of breast milk substitutes. Also includes mention of any hospital policies related to breastfeeding after a positive COVID-19 test including separation of mother and child due to COVID-19 |
| 3G | Other challenges/changes | Other challenges/changes in breastfeeding or feeding of breastmilk substitutes during the COVID-19 pandemic. |
| *4 – Adolescents* | | |
| 4A | Foods preferred | Foods preferred or consumed by adolescents during COVID-19. |
| 4B | Foods avoided/reduced | Foods avoided or consumed in reduced quantity by adolescents during COVID-19. |
| 4C | Beliefs/perceptions | Discussion of adolescents’ attitude, beliefs, and/or perceptions of foods they might consume (e.g., description of foods believed to be healthy or immune boosting or foods believed to be unhealthy). |
| 4D | Physical activity | Discussion of adolescent physical activity including any challenges to staying physically active or changes experienced during the COVID-19 pandemic. |
| 4E | Coping strategies | Types of coping strategies mentioned by adolescents, such as how their parents/families cope, or discussion of any needs for psychosocial support (e.g., adolescent clubs, mental health counsellors/resources). |
| 4F | School attendance | Any discussion of school, reduction in school attendance, school closures, school-led nutrition programs, or other changes in school noted during the COVID-19 pandemic. |
| 4G | Other challenges/changes | Other dietary challenges/changes/stressors adolescents experienced during the COVID-19 pandemic including gaps in psychosocial support. |
| *5 – Health service delivery* | | |
| 5A | Policies/guidelines | Policies/guidelines on COVID-19 (i.e., breastfeeding, masks, social distancing, washing hands/hygiene, nutrition). |
| 5B | Adaptations: health services | Discussion of any adaptations to health services due to COVID-19 (e.g., home visits, reduced hours, giving out masks, working on different days, referrals, and use of traditional birth attendants in both community and facility-based health services) |
| 5C | Access: health services | Discussion of access issues during the COVID-19 pandemic, including access to health services due to fear or transportation costs, health worker strikes, perceptions of services - to access to supplies due to shortages (i.e., PPE, nutrition supplements, and other health equipment) Includes increase/decrease in the number of clients accessing services and includes access to spaces in the health facility such as breastfeeding/postnatal corners. |
| 5D | Other challenges/changes | Other challenges/changes to maternal, infant/young child, and adolescent health services during the COVID-19 pandemic including patient/provider interaction. |
| *6 – Health providers’ roles* | | |
| 6A | Advice/counselling | Advice/counselling health workers say they provide on any particular topic (i.e., health workers are defined as those that work in health, CHV, nutritionist, nurse, etc.) |
| 6B | Activities | Discussion of health providers’ activities performed during COVID-19 - such as referrals, screening, micronutrient supplementation, deworming, and home visits. |
| 6C | Capacity building | Any training received by health providers. |
| *7 – Food vendors* | | |
| 7A | Adaptations: Food, agriculture, trade | Adaptations to agriculture, trade, and the work of food vendors during the COVID-19 pandemic. |
| 7B | Other challenges/changes | Other challenges/changes related to agriculture, trade, and the work of food vendors (e.g., weather, violence). |
| *8 – Effects of COVID-19* | | |
| 8A | Lockdown/ closures/ restricted movement/curfew | Description of lockdown, closures, curfews, restricted movement during COVID-19 and its direct effects including its impacts on transportation for food/mothers. |
| 8B | Food prices and availability | Description of changes in food prices observed or specific foods that were high price, less available, or available in lower quantity, or locally available |
| 8C | Job security | Job loss, inconsistent work, reduced income, or other economic challenges experienced during the COVID-19 pandemic, including loss of income, and closure of personal business. |
| 8D | Food insecurity | Discussion of food insecurity (i.e., not being able to buy food, not having food last a few days, going without food) or decreased access/availability of nutritious foods resulting from job insecurity or other economic factors during the COVID-19 pandemic. |
| 8E | Other challenges/changes | Any other challenges/changes resulting from the COVID-19 pandemic including economic issues and family instability during the COVID-19 pandemic. |
| *9 – COVID-19 support received* | | |
| 9A | Social protection measures | Social protection measures during the COVID-19 pandemic. Includes: Monetary/financial support or food received during the COVID-19 pandemic including support provided by Government, NGOs or other relief organizations either to mothers and/or business/food vendors |
| *10 – Government stakeholders* | | |
| 10A | Stakeholder roles | Description of government stakeholders’ roles, responsibilities, and initiatives led during the COVID-19 pandemic. |
| 10B | Policy/legislation | Discussion of policy and/or legislation related to nutrition, agriculture, and trade during the COVID-19 pandemic. |
| 10C | Stakeholder guidance | Any guidance from stakeholders including guidance related to diet, health, COVID-19, agriculture, and trade. |
